# Harnessing RNA base editing for diverse applications in RNA biology and RNA therapeutics

**DOI:** 10.1007/s44307-025-00063-x

**Published:** 2025-04-08

**Authors:** Hui Luo, Jing Yao, Rui Zhang

**Affiliations:** 1 MOE Key Laboratory of Gene Function and Regulation, Guangdong Province Key Laboratory of Pharmaceutical Functional Genes, State Key Laboratory of Biocontrol, School of Life Sciences, Sun Yat-Sen University, Guangzhou, 510275 PR China; 2 Innovation Center for Evolutionary Synthetic Biology, School of Life Sciences, Sun Yat-Sen University, Guangzhou, 510275 PR China

**Keywords:** RNA editing, ADAR, APOBEC, RNA sensing, Base editing, RNA binding protein

## Abstract

Recent advancements in molecular engineering have established RNA-based technologies as powerful tools for both fundamental research and translational applications. Among the various RNA-based technologies developed, RNA base editing has recently emerged as a groundbreaking advancement. It primarily involves the conversion of adenosine (A) to inosine (I) and cytidine (C) to uridine (U), which are mediated by ADAR and APOBEC enzymes, respectively. RNA base editing has been applied in both biological research and therapeutic contexts. It enables site-directed editing within target transcripts, offering reversible, dose-dependent effects, in contrast to the permanent or heritable changes associated with DNA base editing. Additionally, RNA editing-based profiling of RNA-binding protein (RBP) binding sites facilitates transcriptome-wide mapping of RBP-RNA interactions in specific tissues and at the single-cell level. Furthermore, RNA editing-based sensors have been utilized to express effector proteins in response to specific RNA species. As RNA base editing technologies continue to evolve, we anticipate that they will significantly drive advancements in RNA therapeutics, synthetic biology, and biological research.

## Main

RNA base editing is a naturally occurring phenomenon in eukaryotes involving the post-transcriptional deamination of A-to-I or C-to-U (Gott and Emeson 2000). Notably, inosine is recognized as guanine (G) by the splicing and translational machinery. Among these processes, A-to-I editing is the most prevalent type, occurring ubiquitously across nearly all metazoan cell types. Its primary function is believed to be the evasion of innate immune responses against self double-stranded RNA (dsRNA), with additional roles encompassing the diversification of proteins and RNA species (Li and Church 2013; Eisenberg and Levanon 2018). In contrast, C-to-U editing is predominantly observed in the small intestine, liver, monocytes, and macrophages, and its principal role is yet to be fully elucidated (Pecori et al. 2022).

Recent advancements in high-throughput sequencing technologies have significantly enhanced the identification and mapping of RNA editing events (Ramaswami et al. 2012, 2013; Zhang et al. 2014; Eisenberg 2025; Li et al. 2018). Additionally, the advent of CRISPR-based DNA editing techniques has inspired the development of fusion protein systems that integrate targeting and effector proteins for genome manipulation (Porto et al. 2020). These innovations collectively facilitate the utilization of RNA editing enzymes for a wide array of applications.

In this review, we explore the current applications of RNA editing, including targeted base editing, profiling of RBP binding sites, and RNA-sensor systems. We discuss the underlying engineering principles and challenges associated with these technologies and highlight their potential in both fundamental research and clinical applications.

## Site-directed RNA editing

Site-directed RNA base editing involves the selective editing of specific A or C bases within a target transcript. In contrast to DNA-editing approaches, RNA base editing is transient and reversible, thus reducing the risk of long-lasting inadvertent side-effects (Tao et al. 2023; Zhang et al. 2023). This technique has garnered significant attention due to its ability to correct disease-causing mutations and modulate protein functions, particularly in clinical settings (Gagnidze et al. 2018; Gold et al. 2021).

### Methods of site-directed RNA editing

**Fig. 1:**
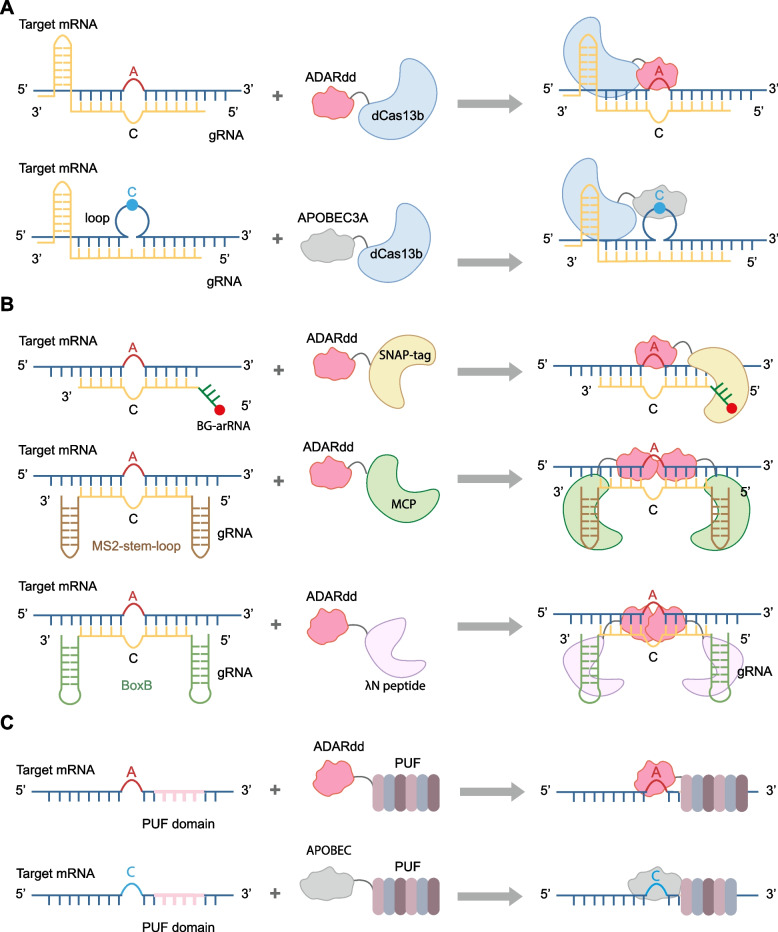
Various RNA base editing platforms with exogenous deaminases. **A** CRISPR-based editing approaches leverage the programmable targeting capability of an inactive dCas13 fused to ADARdd or APOBEC3 A. **B** Other types of fusion protein strategies. The deaminase domain of ADAR2 is fused with an RNA-binding domain that specifically binds to certain sequences, or to a SNAP-tag using click chemistry. The RNA-binding domain can be MCP or λN-peptide. gRNAs composed of targeting region and RNA-binding domain recruiting region. BG-arRNA: Benzyl Guanine ADAR recruiting RNA. **C** Schematic representation of AI/CU-REWIRE-mediated A-to-I/C-to-U RNA editing

The therapeutic potential of ADARs was first explored in 1995 by Woolf et al., who investigated their use in correcting a premature stop codon in dystrophin (Woolf et al. 1995). However, the concept gained renewed attention nearly two decades later by Stafforst and Rosenthal groups (Stafforst and Schneider 2012; Montiel-Gonzalez et al. 2013). Since then, the potential of RNA editing has been increasingly recognized, and today, three major strategies for RNA base editing have been developed (Pfeiffer and Stafforst 2023; Song et al. 2024). The first strategy relies on the ectopic expression of two components: an enzyme and a guide RNA (gRNA). The enzyme can be an ADAR protein or a fusion protein containing the deaminase domain of ADAR (ADARdd) or APOBEC family members. The gRNA is designed to recruit the enzyme to the specific site. Several such methods have been developed, each with its own strengths and limitations. dCas13-based editing approaches take advantage of the programmable targeting of a dCas13 moiety (East-Seletsky et al. 2016; Abudayyeh et al. 2016; Abudayyeh et al. 2017) fused to a catalytically active deaminase domain from ADAR2 (Cox et al. 2017) or APOBEC (Fig. 1A) (Huang et al. 2020). For instance, the REPAIR system employs a gRNA with a spacer (5’homology region) that hybridizes with the substrate and carries an A-C mismatch to direct editing. A direct repeat region at the 3’end of the spacer sequence recruits dCas13b-ADAR fusion protein to the sequence of interest. Additionally, ADAR catalytic domains can be recruited by fusing them to RNA-binding domains that bind to specific sequences or by fusing them to a SNAP-tag via click chemistry (Fig. 1B) (Stafforst and Schneider 2012; Montiel-Gonzalez et al. 2013; Vogel et al. 2014; Azad et al. 2017; Bhakta et al. 2018; Vogel and Stafforst 2019). These gRNAs specifically target the region to be edited and contain a region that recruits the ADAR fusion protein, such as λN-peptide, MCP, or SNAP-tag. Although these binary systems work efficiently in most studies, some inherent obstacles limit their broad applications, especially in therapeutic contexts.

The second system delivers a fusion protein alone (Fig. 1C). For instance, the REWIRE system employs a programmable Pumilio and FBF (PUF) domain that selectively binds RNAs and incorporates a catalytic domain of human ADARs or APOBEC3A enzymes to facilitate the editing of adenosine or cytidine bases (Han et al. 2022). The PUF domain, also known as the Pumilio homology domain, is a conserved protein domain found in RBPs called PUF proteins, which specifically binds to target RNA sequences in mRNAs, primarily regulating translation and mRNA stability (Wang et al. 2018). This domain is characterized by a series of repeating amino acid motifs, typically eight repeats, each of which recognizes a single RNA base, allowing for highly specific RNA binding. Each repeat consists of approximately 36 amino acids. By engineering the programmable PUF domain, the system can efficiently recognize any target sequence of 8–10 nucleotides. For example, CU-REWIRE is developed by combining the PUF domain with the cytosine deaminase APOBEC3A. CU-REWIRE demonstrates editing efficiencies ranging from 20% to 45% at endogenous mRNAs. It also successfully corrects deleterious mutations by targeting two disease-associated T > C mutations in human genes: EZH2 in Weaver syndrome and SCN1A in Dravet syndrome, achieving efficiencies of 21% to 29%. In addition to its ability to edit endogenous mRNAs, CU-REWIRE is also effective in editing viral RNA. When targeting the mRNA of SARS-CoV-2 membrane protein at C246 site, CU-REWIRE achieves a remarkable editing efficiency of approximately 62%. Similarly, AI-REWIRE is developed by combining the PUF domain with the ADARdd.

**Fig. 2:**
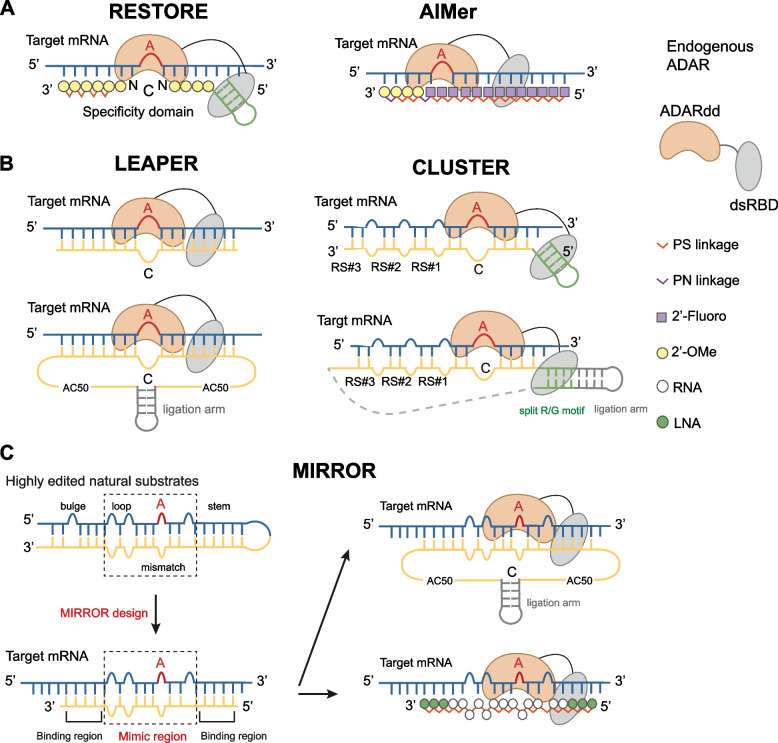
Various RNA base editing platforms with endogenous ADAR recruitment. **A** Chemically modified short ADAR gRNA systems. RESTORE: Recruiting Endogenous ADAR to Specific Transcripts for Oligonucleotide-mediated RNA Editing. AIMers are short and fully chemically modified oligonucleotides with stereopure phosphorothioate (PS) and nitrogen-containing (PN) linkages based on phosphoryl guanidine. **B** Biologically generated long gRNA strategies. LEAPER: Leveraging Endogenous ADAR for Programmable Editing of RNA. CLUSTER gRNAs consist of sequences that bind their target RNA with a cluster of recruitment sequences (RS) dispersed over the target region. Both LEAPER and CLUSTER systems benefit from circular forms, which enhance stability and improve editing efficiency. **C** The MIRROR gRNA design system. MIRROR: Mimicking Inverted Repeats to Recruit ADARs via Engineered Oligoribonucleotide

The third system delivers a single gRNA to recruit endogenous ADARs. The gRNA is either with complex chemical modifications or as a simple long antisense RNA form (Merkle et al. 2019; Qu et al. 2019; Reautschnig et al. 2022). gRNAs designed to utilize ADARs typically comprise a fully complementary specificity domain with an A-C mismatch at the target position to direct editing. Additionally, some gRNAs also incorporate a structured RNA motif to enhance ADAR recruitment. For short, chemically modified gRNAs, those without a structured RNA motif are used in approaches like AIMer (Monian et al. 2022), while those with a structured RNA motif are used in methods such as RESTORE (Merkle et al. 2019) (Fig. 2A). For longer, biologically generated gRNAs, those without a structured RNA motif are employed in approaches like LEAPER (Qu et al. 2019), and those with a structured motif are used in methods such as CLUSTER (Reautschnig et al. 2022) (Fig. 2B). Both strategies are available in circular forms (Katrekar et al. 2022; Yi et al. 2022; Reautschnig et al. 2024), which enhance their stability and thereby increase editing efficiency (Fig. 2B). All these strategies minimally impact the normal A-to-I editing activity of endogenous ADARs and do not trigger significant innate immune activation. The editing efficiency is generally higher for easily edited triplet motifs, such as UAG, and considerably lower for more challenging motifs, such as GAN. Recently, the MIRROR approach has been developed to engineer oligoribonucleotides to recruit ADARs for programmable RNA editing (Sun et al. 2025). MIRROR leverages the rules derived from highly edited natural ADAR substrates to enable rational gRNA design (Fig. 2C). It is applicable to both short chemically synthesized gRNAs with modifications and long biologically generated gRNAs, and has significantly enhanced editing efficiencies, particularly for the non-UAG motifs.

### Applications of RNA base editing

ADAR-mediated RNA base editing offers a safer alternative to genome editing for specific clinical settings. Compared to the first two strategies, the third one holds the greatest therapeutic potential due to its higher safety profile and druggability. As a result, a growing list of biotechs has focused on this strategy for RNA-editing drug development (Table 1).

**Table 1:** Endogenous ADAR-based editors in and approaching the clinic

Sponsor	Properties	Lead indication	Status
Wave Life Sciences/GSK	*SERPINA1*/AAT mRNA editor	Alpha- 1 antitrypsin deficiency	Phase I
Wave Life Sciences/GSK	*PNPLA3* mRNA editor	non-alcoholic fatty liver disease	Preclinical
Wave Life Sciences/GSK	*LDLR* mRNA editor	hypercholesterolemia	Preclinical
ProQR	*B4GALT1* mRNA editor	Cardiovascular disease	Preclinical
ProQR	*NTCP* mRNA editor	Cholestatic diseases	Preclinical
Korro Bio	*SERPINA1*/AAT mRNA editor	Alpha- 1 antitrypsin deficiency	IND
ADARx	*SERPINA1*/AAT mRNA editor	Alpha- 1 antitrypsin deficiency	Preclinical
AIRNA	*SERPINA1*/AAT mRNA editor	Alpha- 1 antitrypsin deficiency	Preclinical
Vico Therapeutics	*MECP2*-R255X mRNA editor	Rett syndrome	Preclinical

A prime example of its therapeutic utility is alpha-1 antitrypsin deficiency (AATD), a monogenic disorder caused by the Z allele in the *SERPINA1* gene (Erion et al. 2025). The Z allele is a G-to-A mutation in the *SERPINA1* CDS, which leads to an E342K missense mutation. This mutation triggers the misfolding and aggregation of the α1-antitrypsin (AAT) protein within hepatocytes. The resulting accumulation not only induces endoplasmic reticulum (ER) stress and progressive liver damage but also reduces the secretion of functional AAT into the systemic circulation, leaving lung tissue vulnerable to neutrophil elastase-mediated degradation—a hallmark of emphysema. ADAR-mediated RNA editing can be applied to correct the mutant *SERPINA1* mRNA, restore proper protein folding, and re-establish protective AAT activity in both hepatic and pulmonary tissues. Several other therapeutic strategies have been explored for AATD. AAT protein supplementation has been used to address lung issues, while siRNA has targeted mutant *SERPINA1* to treat liver damage. DNA base editing has been used to repair hepatocytes, but due to its moderate editing efficiency, it can only restore AAT in a fraction of hepatocytes, thereby solving lung issues but not liver ones. Only RNA editing, which targets the abundant *SERPINA1* mRNA copies in hepatocytes, can simultaneously address both liver and lung issues, offering advantages over all other current strategies. Encouragingly, by the end of 2024, Wave presented promising human data based on their AIMer approach, which uses chemically modified short gRNAs to edit *SERPINA1* mRNAs and correct this mutation.

Beyond mutation correction, RNA editing holds immense promise for modulating gene expression and fine-tuning protein function. For instance, editing mRNA sequences to alter post-translational modification sites, such as phosphorylation, ubiquitination, or glycosylation motifs, can dynamically regulate protein stability, localization, or interaction networks (Diaz Quiroz et al. 2023; Booth et al. 2023). This approach could enable precise control over signaling pathways implicated in cancer, neurodegeneration, or metabolic disorders.

### Limitations and future directions

Depending on the chosen editing systems, various limitations may arise. Several major concerns are associated with the first two overexpression strategies. First, overexpression of ADAR1 or APOBEC has been linked to oncogenic effects in multiple cancers. Second, overexpression can lead to substantial off-target edits, affecting unintended RNA sequences across the transcriptome. Third, the ectopic expression of foreign proteins or protein domains carries the risk of triggering immune responses, potentially resulting in immunogenicity. Therefore, future optimization strategies should focus on reducing off-target effects, potentially through protein engineering to enhance on-target specificity. For example, integrating optogenetic control systems can regulate the timing of RNA base editing, thereby minimizing unwanted effects (Lan et al. 2022).

One of the main limitations of approaches leveraging endogenous ADARs is low editing efficiency, which can reduce their effectiveness. Additionally, as ADAR expression levels vary significantly across different tissues, the applicability of these systems in various tissue types requires further experimental validation. For short chemically modified gRNAs, future optimization efforts may focus on both sequence design and chemical modifications to enhance editing efficiency. For long biologically generated gRNAs, key areas for improvement may include enhancing stability, promoting nuclear retention, optimizing ADAR recruitment, and minimizing bystander editing.

In addition, there are concerns regarding delivery efficiency and the transient nature of RNA editing. However, recent advancements in ligand design and the development of lipid nanoparticle-based delivery systems hold significant promise for overcoming this difficulty (Paunovska et al. 2022). In addition, exosomes, which are nanosized vesicles secreted by cells, are attracting increasing interest as a superior drug delivery system due to their natural stability, biocompatibility, and low immunogenicity. They can cross biological barriers like the blood-brain barrier, enabling targeted delivery to specific cells or tissues while minimizing off-target effects. These innovations could improve the efficiency of RNA base editing in vivo, making it a more viable option for therapeutic applications.

Despite these challenges, site-directed RNA editing remains a highly promising approach. As the field advances, it is poised to play a central role in RNA-based therapeutics, offering the potential to revolutionize disease treatment and pave the way for personalized medicine. With continued innovation and collaboration, RNA base editing could unlock new therapeutic avenues, directly benefiting patients.

## RNA editing-based RBP binding site profiling

RBPs are essential regulators of RNA metabolism throughout the entire RNA life cycle (Gerstberger et al. 2014). They bind to specific RNA sequences, influencing RNA splicing, stability, localization, translation, and function. The human genome alone contains over 2,650 RBPs, underscoring their critical role in cellular processes (Gebauer et al. 2021). To better understand how a particular RBP affects RNA function and fate, it is crucial to identify the specific RNA molecules with which it interacts. Traditionally, methods for detecting RBP targets have relied on cross-linking and immunoprecipitation followed by high-throughput sequencing (CLIP-seq) or similar approaches (Ule et al. 2003; Licatalosi et al. 2008; Hafner et al. 2010; König et al. 2010; Spitzer et al. 2014; Song et al. 2020; Xiang et al. 2024). However, these techniques depend heavily on the availability of suitable antibodies and the efficiency of crosslinking, which limits their applicability. To overcome these challenges, innovative strategies have been developed, such as the creation of RBP-ADAR or RBP-APOBEC fusion proteins, which allow for the detection of RBP binding events through RNA editing. Notably, during RNA sequencing, inosine is recognized as guanosine by reverse transcriptase, resulting in its pairing with cytidine in the cDNA sequence. These approaches enable the identification of RBP binding sites without the need to enrich RBP-RNA complexes via immunoprecipitation, as the resulting mutation signatures can be directly detected using standard RNA-seq techniques, both at the bulk and single-cell level.

### Current approaches

**Fig. 3:**
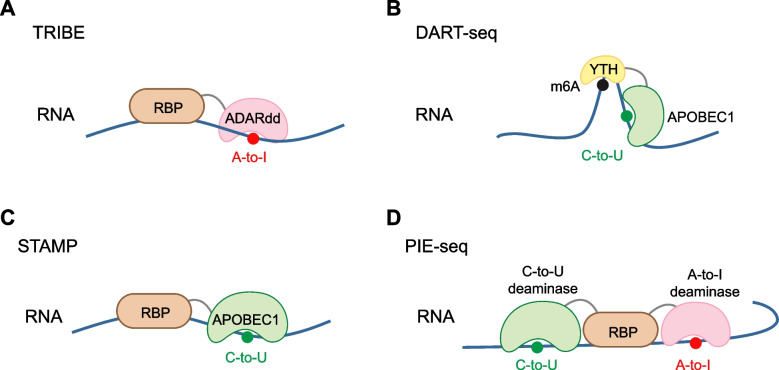
RNA editing-based RBP binding site profiling by different systems. **A** TRIBE: Targets of RNA binding proteins Identified By Editing. **B** DART-seq: Deamination Adjacent to RNA modification Target. **C** STAMP: Surveying Targets by APOBEC-Mediated Profiling. **D** PIE-seq identifies RNA-binding protein targets by dual RNA-deaminase editing and sequencing

In 2016, Rosbash and colleagues introduced the concept of using RBP-deaminase fusion protein to map RBP binding sites. They developed the TRIBE approach (McMahon et al. 2016**) **(Fig. 3A), in which an RBP of interest is fused to ADARdd. The RBP component guides the fusion protein to its natural RNA targets, while the ADARdd introduces A-to-I editing at RBP binding sites. These A-to-I edits are subsequently detected as A-to-G changes in RNA-seq data when compared to the reference transcriptome. This strategy allows the antibody-free detection of RBP binding sites. Two years later, the TRIBE method was enhanced with a hyperactive ADARdd mutation (E488Q) to create HyperTRIBE (Rahman et al. 2018). HyperTRIBE identifies many more editing sites than the original TRIBE method, including many sites also recognized by TRIBE, but at a much higher editing frequency. HyperTRIBE thus more faithfully recapitulates the known binding specificity of the RBP, making it a more effective tool for identifying cell-specific targets of RBPs than traditional methods like CLIP. Additionally, HyperTRIBE requires only minimal amounts of starting material, making it particularly advantageous for studying rare or hard-to-isolate cell types.

Building on the success of TRIBE, novel techniques have been developed that utilize the APOBEC enzyme for C-to-U editing. The DART-seq system, introduced in 2019 (Meyer 2019) (Fig. 3B), is an antibody-free method for detecting m^6^A sites. In DART-seq, the cytosine deaminase APOBEC1 is engineered to form a chimeric protein with the m6A-recognizing YTH domain, enabling the APOBEC1-YTH fusion complex to catalyze site-specific C-to-U conversions proximal to m^6^A modifications. The C-to-U editing events are then detected through RNA-seq. DART-seq detects m^6^A sites with high enrichment in 3'UTRs, near stop codons, and within long internal exons, matching the known distribution of m^6^A. DART-seq can identify thousands of m^6^A sites from as little as 10 ng of total RNA and enable the detection of m^6^A accumulation over time. Furthermore, long-read DART-seq allows for the mapping of the distribution of m^6^A across individual RNA transcripts.

In 2021, an integrated experimental and computational framework called STAMP was developed to map RBP-RNA interactions at isoform-specific and single-cell resolution (Brannan et al. 2021) (Fig. 3C). In this system, the RBP-APOBEC1 fusion protein directs the deaminase module to RNA targets, inducing C-to-U editing near RBP binding sites. The authors further extended this approach to single-cell detection of ribosome-associated RNAs, allowing for simultaneous measurement of gene expression and translation efficiency. Since ADARdd and APOBEC1 have different preferences for RNA substrates (dsRNA vs. ssRNA), each has its advantages and limitations. ADARdd may be less sensitive for mapping RBPs that preferentially bind ssRNA regions, while APOBEC1 may have limited utility for detecting RBP binding sites on dsRNA.

A more recent method, PIE-Seq, was introduced to enhance target RBP binding site detection by using dual deaminases (Ruan et al. 2023) (Fig. 3D). PIE-Seq fuses RBPs to both A-to-I and C-to-U RNA deaminase domains, enabling the dual deaminases to edit adjacent sequences when the PIE-RBP binds its RNA target. These dual edits can be detected using bulk or single-cell RNA-seq. The dual deaminase strategy has the potential to enhance target discovery compared to the single deaminase approach, overcoming biases related to RNA sequence composition and secondary structure preferences inherent in single deaminase methods.

### Limitations and future perspectives

Despite their utility, RNA editing-based RBP binding site profiling methods have several limitations. Notably, they rely on the overexpression of fusion proteins. Since RBPs play essential roles in cellular processes, overexpressing the RBP of interest may lead to non-physiological changes in cell behavior and gene expression. Additionally, an excess of the RBP could result in non-specific binding once the true RNA targets are saturated. The need to induce fusion proteins over 24 hours or more also restricts the ability to capture dynamic RNA binding events. Furthermore, the RNA editing activity of ADARs/APOBECs may impact mRNA stability, introducing potential biases in the profiling results.

As a novel strategy to map RBP binding sites, RNA editing-based methods offer several advantages and present opportunities for future applications and optimizations. By utilizing cell-type-specific promoters, RNA editing-based methods can be adapted to map RBP binding in a cell-type-specific context, which is not achievable using CLIP-seq-based techniques. Additionally, targeted genomic integration of editing modules in cell or animal models could circumvent the overexpression issue, enabling more faithful mapping of binding sites both in vitro and in vivo. This strategy would be particularly powerful for tracing RNA-protein interactions in previously inaccessible contexts, offering insights into isoform-specific binding and providing tissue- and cell-type-specific profiling in development and disease. Future innovations, such as incorporating light- or chemical-activatable domains to control editing spatially and temporally, could mitigate potential functional effects introduced by the RNA editing events, especially for in vivo applications.

## RNA editing-based sensors

RNAs are essential messengers that convey genetic instructions tailored to specific cell types and states. With the increasing availability of single-cell and spatial transcriptomics, RNA signatures have become a promising foundation for targeting cells in diverse biological contexts (Longo et al. 2021; Elmentaite et al. 2022; Piwecka et al. 2023). Moreover, monitoring and manipulating RNA across various cell types is crucial for understanding its role in tissue architecture and function in vivo, and is also vital for the precise diagnosis and treatment of many human diseases. RNA sensors, capable of detecting and modulating specific RNA molecules, facilitate these studies. Several RNA-sensing technologies have been developed, including methods for detecting miRNAs and those designed to bind specific mRNAs to alter the desired payload mRNA structures, inducing internal ribosome entry sites for the expression of the payload protein (Xie et al. 2011; Zhao et al. 2022). However, these tools are often limited to miRNAs or suffer from reduced specificity and efficiency. A recent, elegant platform has been introduced that leverages RNA editing mechanism to generate a desired payload protein upon the detection of a specific RNA, marking a significant advancement in the field of RNA sensing.

### Principle of ADAR editing-based RNA sensors

**Fig. 4:**
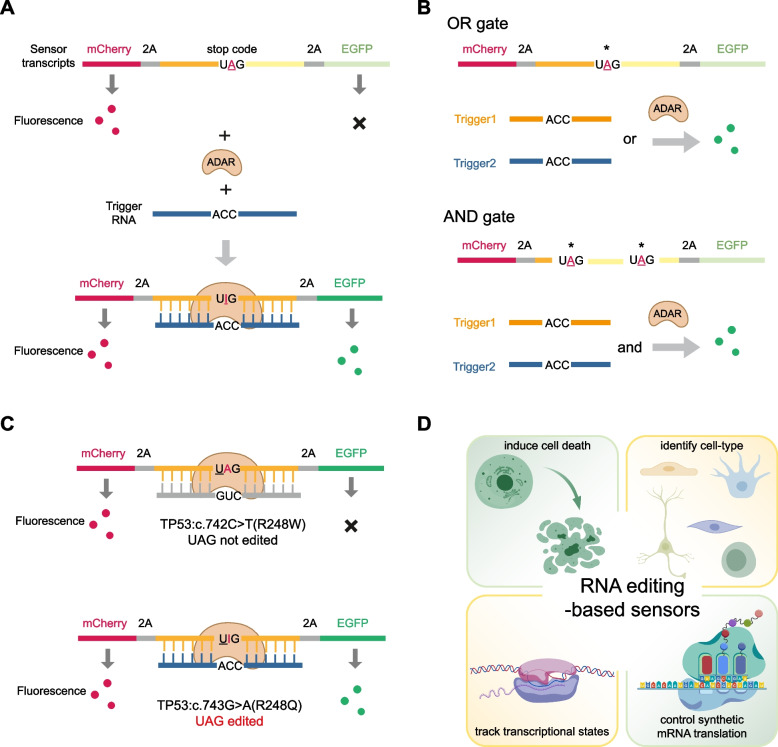
RNA editing-based sensor techniques. **A** The principle of ADAR editing-based RNA sensor. The sensor transcripts contain a domain with a stop codon, functioning as a translation switch. Downstream of this stop codon, in-frame, is a sequence encoding the T2A peptide and the payload protein. In cells expressing the target RNA (i.e., the trigger RNA), the sensor hybridizes with the trigger RNA, forming a dsRNA that recruits ADARs. ADARs edit the stop codon, converting it into a UGG codon, thus enabling translational readthrough and activating translation of the downstream payload. **B** RNA editing-based sensor integrates multiple inputs using OR and AND logic gates. **C** RNA editing-based sensor can be used to distinguish single base changes. **D** RNA editing-based sensors have versatile applications, including tracking transcriptional states, inducing RNA-sensing cell death, cell type identification, and controlling synthetic mRNA translation

In 2022, Huang and colleagues introduced the ADAR editing-based RNA sensor called CellREADR (Qian et al. 2022) (Fig. 4A). In this system, the 5’ region contains a sensor domain of approximately 300 nt that is complementary to a specific cellular RNA, enabling sequence-specific base-pairing for detection. This sensor domain incorporates one or more ADAR-editable stop codons, acting as a translation switch. This domain is referred to as the sense-edit-switch RNA (sesRNA). Downstream of the sesRNA, in-frame with the stop codon, is a sequence encoding the self-cleaving peptide T2A, followed by an effector RNA (efRNA) region that encodes various payload proteins. In cells expressing the target RNA, the sesRNA anneals to the target RNA, generating a double-stranded RNA (dsRNA) structure that recruits ADARs to form an editing complex. At the specified stop codon, ADARs catalyze A into I. This A-to-I conversion, functionally equivalent to an A-to-G substitution, reprograms the UAG stop codon into a UGG codon, thereby encoding tryptophan and enabling translational readthrough, activating translation of the efRNA. The in-frame translation produces a fusion protein consisting of an N-terminal peptide, T2A, and a C-terminal payload protein, which undergoes autonomous cleavage at the T2A site to yield the functional payload protein in its active form. Using this system, researchers successfully recorded and controlled neuronal function in mice.

Building on the same idea, Gao and colleagues introduced RADAR, a modular, programmable system for RNA sensing (Kaseniit et al. 2023). RADAR offers capabilities for cell classification and integrates multiple inputs using OR and AND logic gates (Fig. 4B). Moreover, Gao et al. applied RADAR to distinguish between two common oncogenic mutations of *TP53*, each associated with different invasive traits in cancer cells, demonstrating its ability to distinguish single base changes (Fig. 4C).

Similarly, a platform called RADARS was developed by Omar O. Abudayyeh and colleagues. This platform utilizes the same RNA editing mechanism (Jiang et al. 2023). The RADARS system was engineered to achieve high signal-to-noise ratios. Through systematic sensor optimization, they achieved a 277-fold improvement in activation and engineered RADARS to deliver a range of payload proteins, including luciferases, fluorescent proteins, recombinases, and caspases. RADARS exhibits functionality whether delivered as a DNA construct for expression or as a synthetic mRNA. It is also compatible with both exogenously introduced and endogenously expressed ADAR enzymes. This versatility enables RADARS to be applied in a variety of contexts, including tracking transcriptional states, inducing RNA-sensing-mediated cell death, identifying cell types, and controlling synthetic mRNA translation (Fig. 4D). For example, RADARS was evaluated for its sensitivity in detecting endogenous gene expression changes. Using siRNA-mediated knockdown, RADARS successfully tracked transcriptional downregulation across six endogenous genes, demonstrating a high Pearson correlation with qPCR-measured transcript levels. RADARS was also tested for detecting gene upregulation in a heat-shock model targeting HSP70 in HeLa cells, showing strong agreement with qPCR results. To demonstrate RNA-sensing-mediated cell death, RADARS was designed to target *SERPINA1*, a liver-specific gene, in combination with an iCaspase9 cargo to induce cell-type-specific apoptosis. Co-transfection of the SERPINA1-iCaspase9 RADARS construct with ADARp150 into A549, HeLa, and HepG2 cells selectively killed HepG2 cells, which express *SERPINA1*, while minimizing toxicity in non-target cell types. Moreover, synthetic mRNA RADARS significantly activated luciferase in the presence of the target of interest, further highlighting its functional versatility.

### Limitations and future perspectives

One major limitation of current systems is the low editing efficiency of sesRNA. While low payload expression is acceptable for applications like marking cell types with fluorescent proteins, it severely limits other applications, such as cell type-specific expression of therapeutic proteins. Some strategies, such as overexpressing ADAR, have been developed but are subject to safety concerns. Future directions may focus on optimizing the sensor domain for better editing efficiency, and strategies developed for RNA-editing gRNAs could be adapted for this purpose. Another potential strategy is to increase payload stability, such as using circular RNA forms to enhance stability.

In contrast to previous methods, these ADAR editing-based RNA sensors offer rapid design and a wide range of flexible applications. They feature a reprogrammable guide region that can be targeted using base-pairing rules, along with a modular downstream payload for protein translation. Together, these sensors lay the foundation for a versatile, reprogrammable biological sensor platform with broad potential in biomedical research, diagnostics, and therapeutics.

## Conclusion

In conclusion, RNA editing has evolved into versatile and powerful tools with a broad range of applications in molecular biology. As advancements in RNA editing technologies and delivery systems continue to progress, it is anticipated that these innovations will significantly accelerate progress in RNA biology, RNA therapeutics, and cell biology.

## Data Availability

No datasets were generated or analyzed during the current study.
